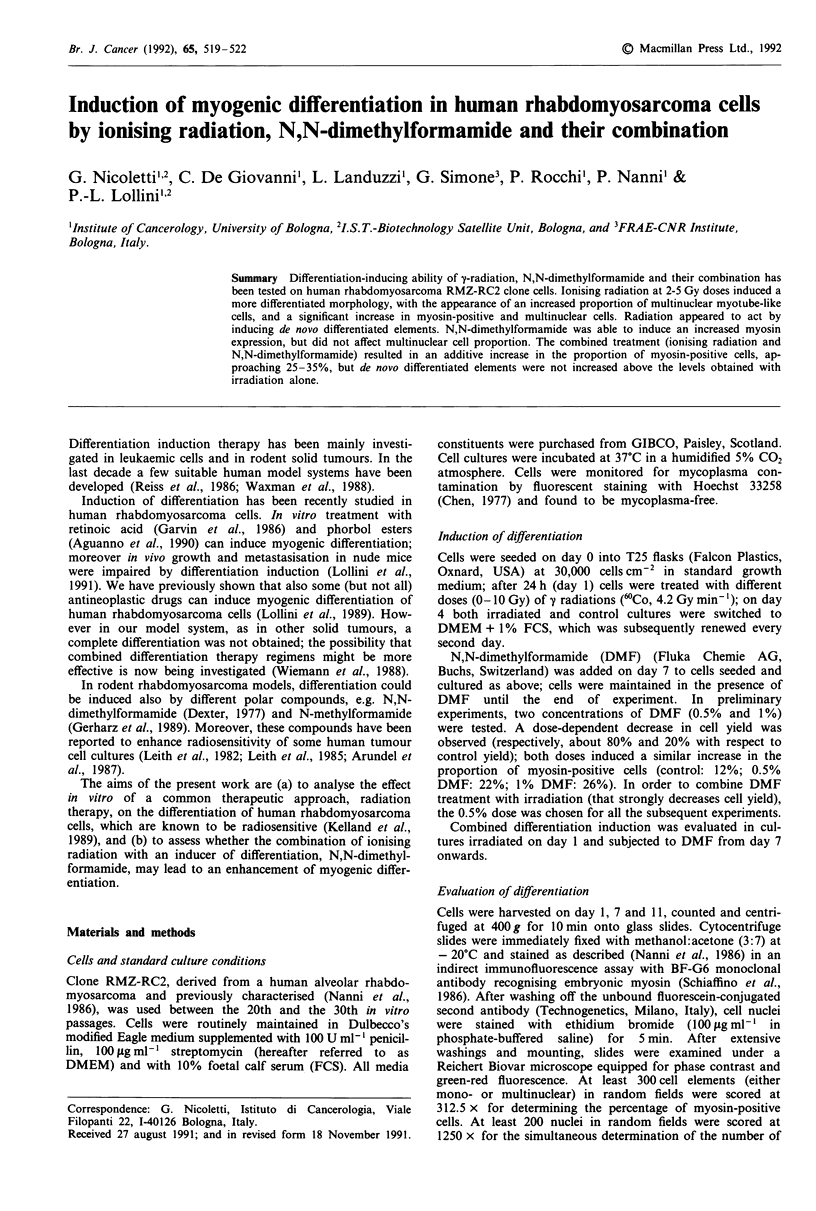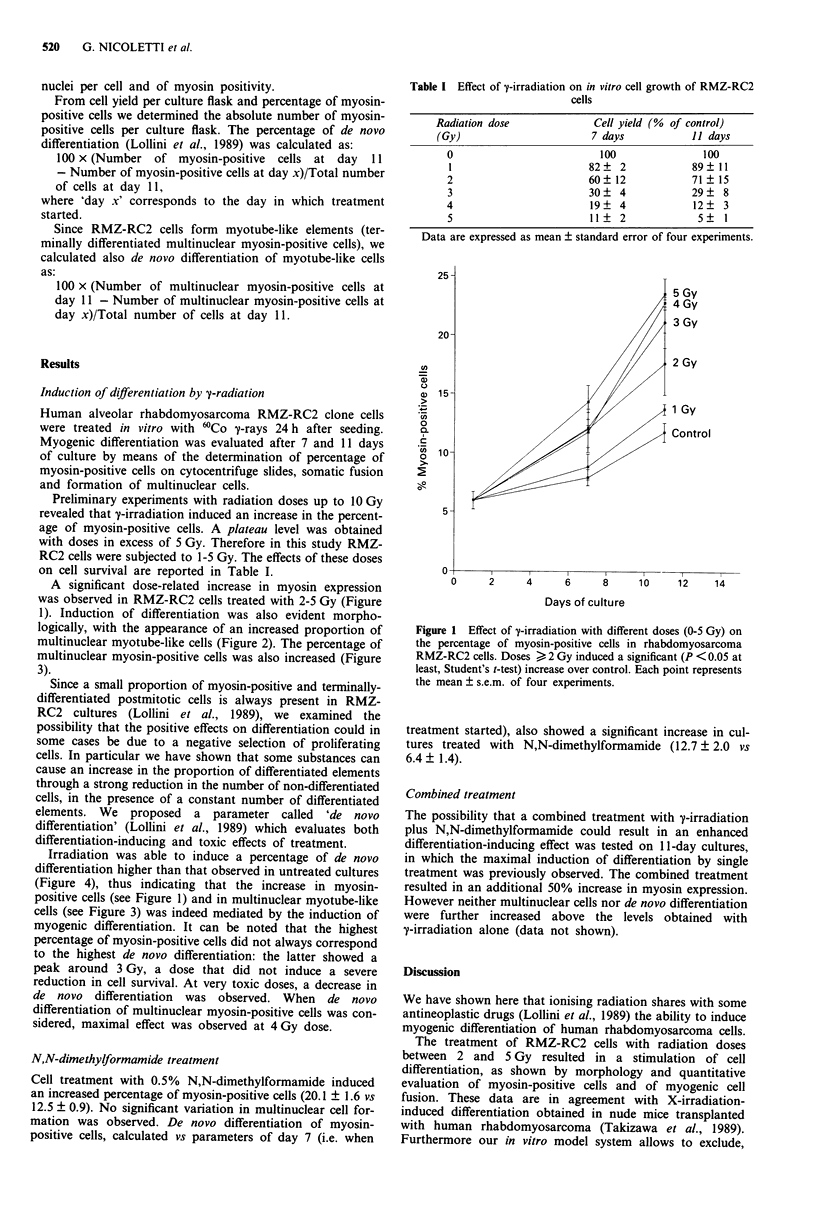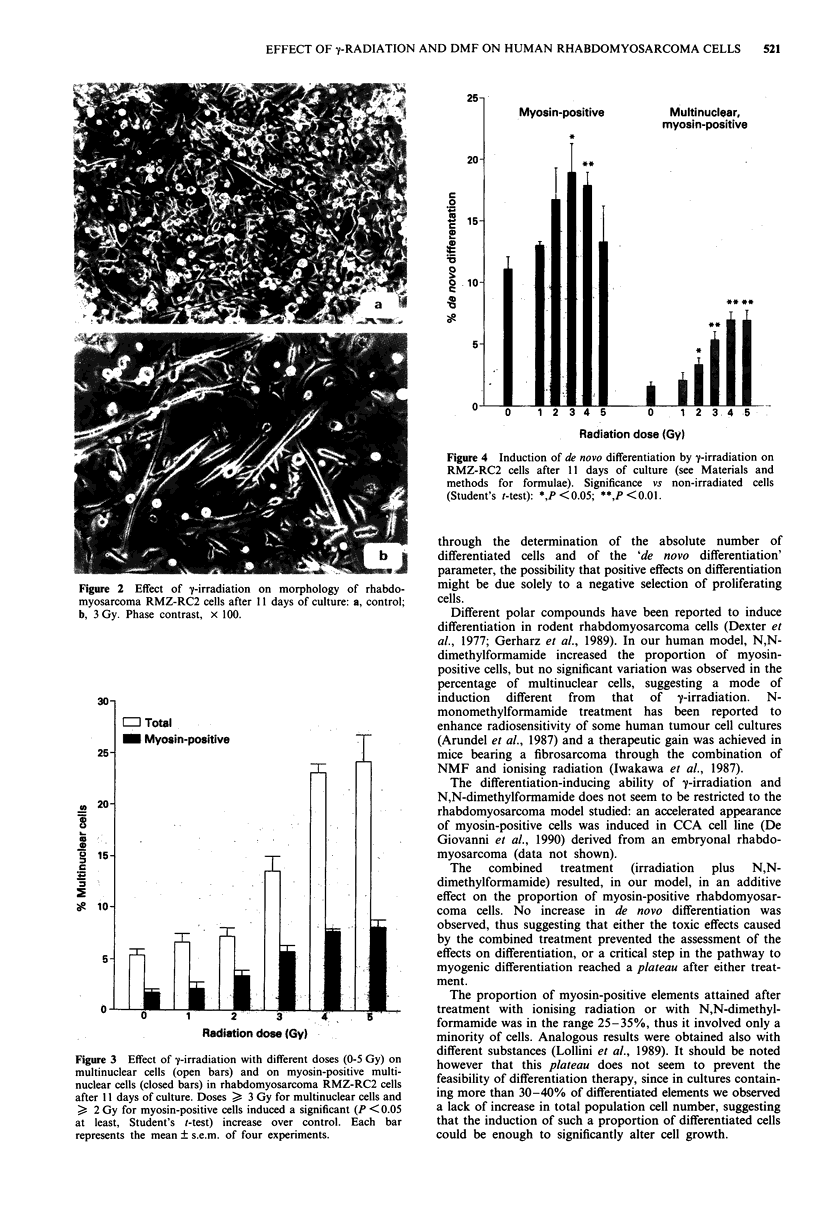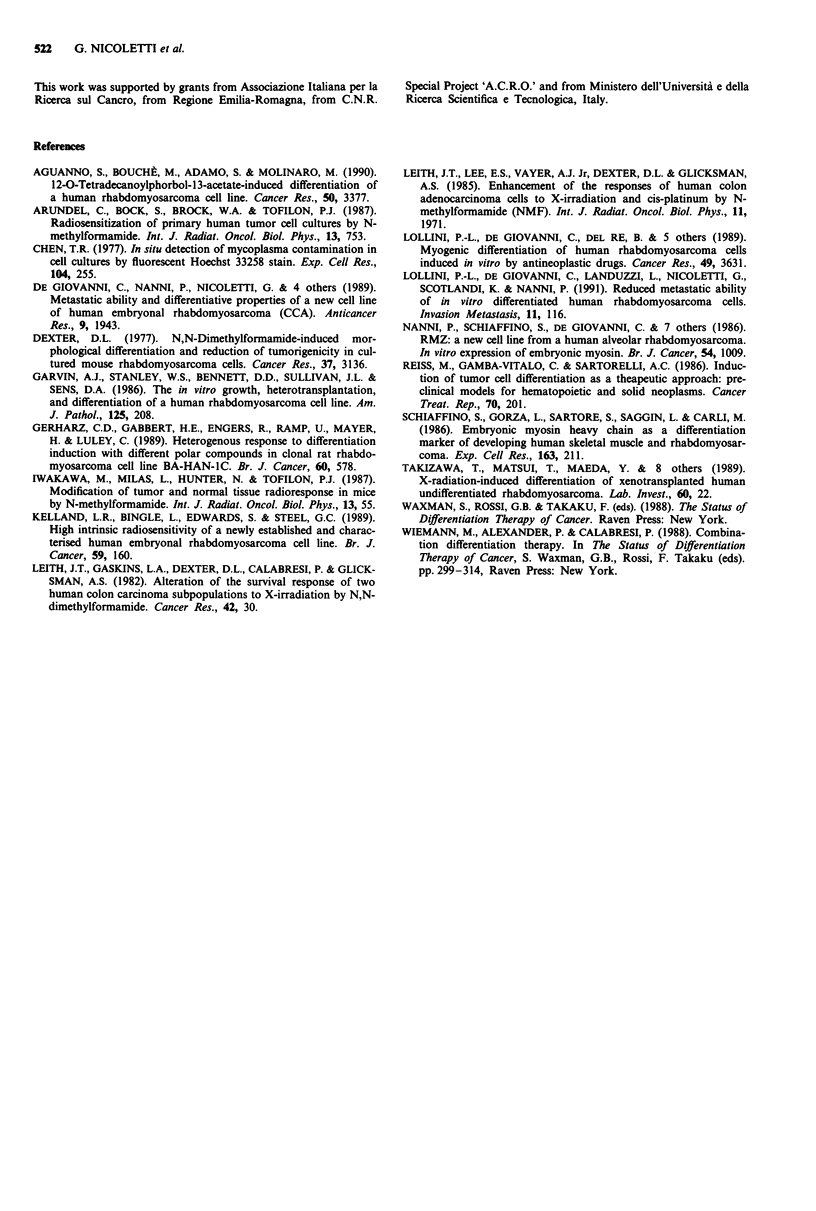# Induction of myogenic differentiation in human rhabdomyosarcoma cells by ionising radiation, N,N-dimethylformamide and their combination.

**DOI:** 10.1038/bjc.1992.107

**Published:** 1992-04

**Authors:** G. Nicoletti, C. De Giovanni, L. Landuzzi, G. Simone, P. Rocchi, P. Nanni, P. L. Lollini

**Affiliations:** Institute of Cancerology, University of Bologna, Italy.

## Abstract

**Images:**


Br. J. Cancer (1992), 65, 519–522 © Macmillan Press Ltd., 1992

### Induction of myogenic differentiation in human rhabdomyosarcoma cells by ionising radiation, N,N-dimethylformamide and their combination

G. Nicoletti[1,2], C. De Giovanni[1], L. Landuzzi[1], G. Simone[3], P. Rocchi[1], P. Nanni[1] & P.-L. Lollini[1,2]

[1]*Institute of Cancerology, University of Bologna,* [2]*I.S.T.-Biotechnology Satellite Unit, Bologna, and* [3]*FRAE-CNR Institute, Bologna, Italy.*

**Summary** Differentiation-inducing ability of γ-radiation, N,N-dimethylformamide and their combination has been tested on human rhabdomyosarcoma RMZ-RC2 clone cells. Ionising radiation at 2-5 Gy doses induced a more differentiated morphology, with the appearance of an increased proportion of multinuclear myotube-like cells, and a significant increase in myosin-positive and multinuclear cells. Radiation appeared to act by inducing *de novo* differentiated elements. N,N-dimethylformamide was able to induce an increased myosin expression, but did not affect multinuclear cell proportion. The combined treatment (ionising radiation and N,N-dimethylformamide) resulted in an additive increase in the proportion of myosin-positive cells, approaching 25–35%, but *de novo* differentiated elements were not increased above the levels obtained with irradiation alone.

Differentiation induction therapy has been mainly investigated in leukaemic cells and in rodent solid tumours. In the last decade a few suitable human model systems have been developed (Reiss *et al.*, 1986; Waxman *et al.*, 1988).

Induction of differentiation has been recently studied in human rhabdomyosarcoma cells. *In vitro* treatment with retinoic acid (Garvin *et al.*, 1986) and phorbol esters (Aguanno *et al.*, 1990) can induce myogenic differentiation; moreover *in vivo* growth and metastasisation in nude mice were impaired by differentiation induction (Lollini *et al.*, 1991). We have previously shown that also some (but not all) antineoplastic drugs can induce myogenic differentiation of human rhabdomyosarcoma cells (Lollini *et al.*, 1989). However in our model system, as in other solid tumours, a complete differentiation was not obtained; the possibility that combined differentiation therapy regimens might be more effective is now being investigated (Wiemann *et al.*, 1988).

In rodent rhabdomyosarcoma models, differentiation could be induced also by different polar compounds, e.g. N,N-dimethylformamide (Dexter, 1977) and N-methylformamide (Gerharz *et al.*, 1989). Moreover, these compounds have been reported to enhance radiosensitivity of some human tumour cell cultures (Leith *et al.*, 1982; Leith *et al.*, 1985; Arundel *et al.*, 1987).

The aims of the present work are (a) to analyse the effect *in vitro* of a common therapeutic approach, radiation therapy, on the differentiation of human rhabdomyosarcoma cells, which are known to be radiosensitive (Kelland *et al.*, 1989), and (b) to assess whether the combination of ionising radiation with an inducer of differentiation, N,N-dimethylformamide, may lead to an enhancement of myogenic differentiation.

### Materials and methods

### *Cells and standard culture conditions*

Clone RMZ-RC2, derived from a human alveolar rhabdomyosarcoma and previously characterised (Nanni *et al.*, 1986), was used between the 20th and the 30th *in vitro* passages. Cells were routinely maintained in Dulbecco's modified Eagle medium supplemented with 100 U $ml^{-1}$ penicillin, 100 $\mu g$ $ml^{-1}$ streptomycin (hereafter referred to as DMEM) and with 10% foetal calf serum (FCS). All media constituents were purchased from GIBCO, Paisley, Scotland. Cell cultures were incubated at 37°C in a humidified 5% $CO_2$ atmosphere. Cells were monitored for mycoplasma contamination by fluorescent staining with Hoechst 33258 (Chen, 1977) and found to be mycoplasma-free.

Correspondence: G. Nicoletti, Istituto di Cancerologia, Viale Filopanti 22, I-40126 Bologna, Italy.
Received 27 august 1991; and in revised form 18 November 1991.

### *Induction of differentiation*

Cells were seeded on day 0 into T25 flasks (Falcon Plastics, Oxnard, USA) at 30,000 cells $cm^{-2}$ in standard growth medium; after 24 h (day 1) cells were treated with different doses (0–10 Gy) of γ radiations ($^{60}Co$, 4.2 Gy $min^{-1}$); on day 4 both irradiated and control cultures were switched to DMEM + 1% FCS, which was subsequently renewed every second day.

N,N-dimethylformamide (DMF) (Fluka Chemie AG, Buchs, Switzerland) was added on day 7 to cells seeded and cultured as above; cells were maintained in the presence of DMF until the end of experiment. In preliminary experiments, two concentrations of DMF (0.5% and 1%) were tested. A dose-dependent decrease in cell yield was observed (respectively, about 80% and 20% with respect to control yield); both doses induced a similar increase in the proportion of myosin-positive cells (control: 12%; 0.5% DMF: 22%; 1% DMF: 26%). In order to combine DMF treatment with irradiation (that strongly decreases cell yield), the 0.5% dose was chosen for all the subsequent experiments.

Combined differentiation induction was evaluated in cultures irradiated on day 1 and subjected to DMF from day 7 onwards.

### *Evaluation of differentiation*

Cells were harvested on day 1, 7 and 11, counted and centrifuged at 400 *g* for 10 min onto glass slides. Cytocentrifuge slides were immediately fixed with methanol:acetone (3:7) at − 20°C and stained as described (Nanni *et al.*, 1986) in an indirect immunofluorescence assay with BF-G6 monoclonal antibody recognising embryonic myosin (Schiaffino *et al.*, 1986). After washing off the unbound fluorescein-conjugated second antibody (Technogenetics, Milano, Italy), cell nuclei were stained with ethidium bromide (100 $\mu g$ $ml^{-1}$ in phosphate-buffered saline) for 5 min. After extensive washings and mounting, slides were examined under a Reichert Biovar microscope equipped for phase contrast and green-red fluorescence. At least 300 cell elements (either mono- or multinuclear) in random fields were scored at 312.5 × for determining the percentage of myosin-positive cells. At least 200 nuclei in random fields were scored at 1250 × for the simultaneous determination of the number of nuclei per cell and of myosin positivity.

From cell yield per culture flask and percentage of myosin-positive cells we determined the absolute number of myosin-positive cells per culture flask. The percentage of *de novo* differentiation (Lollini *et al.*, 1989) was calculated as:

100 × (Number of myosin-positive cells at day 11 − Number of myosin-positive cells at day *x*)/Total number of cells at day 11, where 'day *x*' corresponds to the day in which treatment started.

Since RMZ-RC2 cells form myotube-like elements (terminally differentiated multinuclear myosin-positive cells), we calculated also *de novo* differentiation of myotube-like cells as:

100 × (Number of multinuclear myosin-positive cells at day 11 − Number of multinuclear myosin-positive cells at day *x*)/Total number of cells at day 11.

### Results

### *Induction of differentiation by γ-radiation*

Human alveolar rhabdomyosarcoma RMZ-RC2 clone cells were treated *in vitro* with $^{60}$Co γ-rays 24 h after seeding. Myogenic differentiation was evaluated after 7 and 11 days of culture by means of the determination of percentage of myosin-positive cells on cytocentrifuge slides, somatic fusion and formation of multinuclear cells.

Preliminary experiments with radiation doses up to 10 Gy revealed that γ-irradiation induced an increase in the percentage of myosin-positive cells. A *plateau* level was obtained with doses in excess of 5 Gy. Therefore in this study RMZ-RC2 cells were subjected to 1-5 Gy. The effects of these doses on cell survival are reported in Table I.

A significant dose-related increase in myosin expression was observed in RMZ-RC2 cells treated with 2-5 Gy (Figure 1). Induction of differentiation was also evident morphologically, with the appearance of an increased proportion of multinuclear myotube-like cells (Figure 2). The percentage of multinuclear myosin-positive cells was also increased (Figure 3).

Since a small proportion of myosin-positive and terminally-differentiated postmitotic cells is always present in RMZ-RC2 cultures (Lollini *et al.*, 1989), we examined the possibility that the positive effects on differentiation could in some cases be due to a negative selection of proliferating cells. In particular we have shown that some substances can cause an increase in the proportion of differentiated elements through a strong reduction in the number of non-differentiated cells, in the presence of a constant number of differentiated elements. We proposed a parameter called '*de novo* differentiation' (Lollini *et al.*, 1989) which evaluates both differentiation-inducing and toxic effects of treatment.

Irradiation was able to induce a percentage of *de novo* differentiation higher than that observed in untreated cultures (Figure 4), thus indicating that the increase in myosin-positive cells (see Figure 1) and in multinuclear myotube-like cells (see Figure 3) was indeed mediated by the induction of myogenic differentiation. It can be noted that the highest percentage of myosin-positive cells did not always correspond to the highest *de novo* differentiation: the latter showed a peak around 3 Gy, a dose that did not induce a severe reduction in cell survival. At very toxic doses, a decrease in *de novo* differentiation was observed. When *de novo* differentiation of multinuclear myosin-positive cells was considered, maximal effect was observed at 4 Gy dose.

### *N,N-dimethylformamide treatment*

Cell treatment with 0.5% N,N-dimethylformamide induced an increased percentage of myosin-positive cells (20.1 ± 1.6 *vs* 12.5 ± 0.9). No significant variation in multinuclear cell formation was observed. *De novo* differentiation of myosin-positive cells, calculated *vs* parameters of day 7 (i.e. when treatment started), also showed a significant increase in cultures treated with N,N-dimethylformamide (12.7 ± 2.0 *vs* 6.4 ± 1.4).

**Table I** Effect of γ-irradiation on *in vitro* cell growth of RMZ-RC2 cells

| *Radiation dose (Gy)* | *Cell yield (% of control) 7 days* | *11 days* |
|---|---|---|
| 0 | 100 | 100 |
| 1 | 82 ± 2 | 89 ± 11 |
| 2 | 60 ± 12 | 71 ± 15 |
| 3 | 30 ± 4 | 29 ± 8 |
| 4 | 19 ± 4 | 12 ± 3 |
| 5 | 11 ± 2 | 5 ± 1 |

Data are expressed as mean ± standard error of four experiments.

**Figure 1** Effect of γ-irradiation with different doses (0-5 Gy) on the percentage of myosin-positive cells in rhabdomyosarcoma RMZ-RC2 cells. Doses ≥2 Gy induced a significant ($P < 0.05$ at least, Student's *t*-test) increase over control. Each point represents the mean ± s.e.m. of four experiments.

### *Combined treatment*

The possibility that a combined treatment with γ-irradiation plus N,N-dimethylformamide could result in an enhanced differentiation-inducing effect was tested on 11-day cultures, in which the maximal induction of differentiation by single treatment was previously observed. The combined treatment resulted in an additional 50% increase in myosin expression. However neither multinuclear cells nor *de novo* differentiation were further increased above the levels obtained with γ-irradiation alone (data not shown).

### Discussion

We have shown here that ionising radiation shares with some antineoplastic drugs (Lollini *et al.*, 1989) the ability to induce myogenic differentiation of human rhabdomyosarcoma cells.

The treatment of RMZ-RC2 cells with radiation doses between 2 and 5 Gy resulted in a stimulation of cell differentiation, as shown by morphology and quantitative evaluation of myosin-positive cells and of myogenic cell fusion. These data are in agreement with X-irradiation-induced differentiation obtained in nude mice transplanted with human rhabdomyosarcoma (Takizawa *et al.*, 1989). Furthermore our *in vitro* model system allows to exclude,

**Figure 2** Effect of γ-irradiation on morphology of rhabdomyosarcoma RMZ-RC2 cells after 11 days of culture: **a**, control; **b**, 3 Gy. Phase contrast, × 100.

**Figure 3** Effect of γ-irradiation with different doses (0-5 Gy) on multinuclear cells (open bars) and on myosin-positive multinuclear cells (closed bars) in rhabdomyosarcoma RMZ-RC2 cells after 11 days of culture. Doses ⩾ 3 Gy for multinuclear cells and ⩾ 2 Gy for myosin-positive cells induced a significant ($P < 0.05$ at least, Student's *t*-test) increase over control. Each bar represents the mean ± s.e.m. of four experiments.

**Figure 4** Induction of *de novo* differentiation by γ-irradiation on RMZ-RC2 cells after 11 days of culture (see Materials and methods for formulae). Significance *vs* non-irradiated cells (Student's *t*-test): *,$P < 0.05$; **,$P < 0.01$.

through the determination of the absolute number of differentiated cells and of the '*de novo* differentiation' parameter, the possibility that positive effects on differentiation might be due solely to a negative selection of proliferating cells.

Different polar compounds have been reported to induce differentiation in rodent rhabdomyosarcoma cells (Dexter *et al.*, 1977; Gerharz *et al.*, 1989). In our human model, N,N-dimethylformamide increased the proportion of myosin-positive cells, but no significant variation was observed in the percentage of multinuclear cells, suggesting a mode of induction different from that of γ-irradiation. N-monomethylformamide treatment has been reported to enhance radiosensitivity of some human tumour cell cultures (Arundel *et al.*, 1987) and a therapeutic gain was achieved in mice bearing a fibrosarcoma through the combination of NMF and ionising radiation (Iwakawa *et al.*, 1987).

The differentiation-inducing ability of γ-irradiation and N,N-dimethylformamide does not seem to be restricted to the rhabdomyosarcoma model studied: an accelerated appearance of myosin-positive cells was induced in CCA cell line (De Giovanni *et al.*, 1990) derived from an embryonal rhabdomyosarcoma (data not shown).

The combined treatment (irradiation plus N,N-dimethylformamide) resulted, in our model, in an additive effect on the proportion of myosin-positive rhabdomyosarcoma cells. No increase in *de novo* differentiation was observed, thus suggesting that either the toxic effects caused by the combined treatment prevented the assessment of the effects on differentiation, or a critical step in the pathway to myogenic differentiation reached a *plateau* after either treatment.

The proportion of myosin-positive elements attained after treatment with ionising radiation or with N,N-dimethylformamide was in the range 25–35%, thus it involved only a minority of cells. Analogous results were obtained also with different substances (Lollini *et al.*, 1989). It should be noted however that this *plateau* does not seem to prevent the feasibility of differentiation therapy, since in cultures containing more than 30–40% of differentiated elements we observed a lack of increase in total population cell number, suggesting that the induction of such a proportion of differentiated cells could be enough to significantly alter cell growth.

This work was supported by grants from Associazione Italiana per la Ricerca sul Cancro, from Regione Emilia-Romagna, from C.N.R. Special Project 'A.C.R.O.' and from Ministero dell'Università e della Ricerca Scientifica e Tecnologica, Italy.

### References

AGUANNO, S., BOUCHÈ, M., ADAMO, S. & MOLINARO, M. (1990). 12-O-Tetradecanoylphorbol-13-acetate-induced differentiation of a human rhabdomyosarcoma cell line. *Cancer Res.*, **50**, 3377.

ARUNDEL, C., BOCK, S., BROCK, W.A. & TOFILON, P.J. (1987). Radiosensitization of primary human tumor cell cultures by N-methylformamide. *Int. J. Radiat. Oncol. Biol. Phys.*, **13**, 753.

CHEN, T.R. (1977). *In situ* detection of mycoplasma contamination in cell cultures by fluorescent Hoechst 33258 stain. *Exp. Cell Res.*, **104**, 255.

DE GIOVANNI, C., NANNI, P., NICOLETTI, G. & 4 others (1989). Metastatic ability and differentiative properties of a new cell line of human embryonal rhabdomyosarcoma (CCA). *Anticancer Res.*, **9**, 1943.

DEXTER, D.L. (1977). N,N-Dimethylformamide-induced morphological differentiation and reduction of tumorigenicity in cultured mouse rhabdomyosarcoma cells. *Cancer Res.*, **37**, 3136.

GARVIN, A.J., STANLEY, W.S., BENNETT, D.D., SULLIVAN, J.L. & SENS, D.A. (1986). The *in vitro* growth, heterotransplantation, and differentiation of a human rhabdomyosarcoma cell line. *Am. J. Pathol.*, **125**, 208.

GERHARZ, C.D., GABBERT, H.E., ENGERS, R., RAMP, U., MAYER, H. & LULEY, C. (1989). Heterogenous response to differentiation induction with different polar compounds in clonal rat rhabdomyosarcoma cell line BA-HAN-1C. *Br. J. Cancer*, **60**, 578.

IWAKAWA, M., MILAS, L., HUNTER, N. & TOFILON, P.J. (1987). Modification of tumor and normal tissue radioresponse in mice by N-methylformamide. *Int. J. Radiat. Oncol. Biol. Phys.*, **13**, 55.

KELLAND, L.R., BINGLE, L., EDWARDS, S. & STEEL, G.C. (1989). High intrinsic radiosensitivity of a newly established and characterised human embryonal rhabdomyosarcoma cell line. *Br. J. Cancer*, **59**, 160.

LEITH, J.T., GASKINS, L.A., DEXTER, D.L., CALABRESI, P. & GLICKSMAN, A.S. (1982). Alteration of the survival response of two human colon carcinoma subpopulations to X-irradiation by N,N-dimethylformamide. *Cancer Res.*, **42**, 30.

LEITH, J.T., LEE, E.S., VAYER, A.J. Jr, DEXTER, D.L. & GLICKSMAN, A.S. (1985). Enhancement of the responses of human colon adenocarcinoma cells to X-irradiation and cis-platinum by N-methylformamide (NMF). *Int. J. Radiat. Oncol. Biol. Phys.*, **11**, 1971.

LOLLINI, P.-L., DE GIOVANNI, C., DEL RE, B. & 5 others (1989). Myogenic differentiation of human rhabdomyosarcoma cells induced *in vitro* by antineoplastic drugs. *Cancer Res.*, **49**, 3631.

LOLLINI, P.-L., DE GIOVANNI, C., LANDUZZI, L., NICOLETTI, G., SCOTLANDI, K. & NANNI, P. (1991). Reduced metastatic ability of *in vitro* differentiated human rhabdomyosarcoma cells. *Invasion Metastasis*, **11**, 116.

NANNI, P., SCHIAFFINO, S., DE GIOVANNI, C. & 7 others (1986). RMZ: a new cell line from a human alveolar rhabdomyosarcoma. *In vitro* expression of embryonic myosin. *Br. J. Cancer*, **54**, 1009.

REISS, M., GAMBA-VITALO, C. & SARTORELLI, A.C. (1986). Induction of tumor cell differentiation as a theapeutic approach: preclinical models for hematopoietic and solid neoplasms. *Cancer Treat. Rep.*, **70**, 201.

SCHIAFFINO, S., GORZA, L., SARTORE, S., SAGGIN, L. & CARLI, M. (1986). Embryonic myosin heavy chain as a differentiation marker of developing human skeletal muscle and rhabdomyosarcoma. *Exp. Cell Res.*, **163**, 211.

TAKIZAWA, T., MATSUI, T., MAEDA, Y. & 8 others (1989). X-radiation-induced differentiation of xenotransplanted human undifferentiated rhabdomyosarcoma. *Lab. Invest.*, **60**, 22.

WAXMAN, S., ROSSI, G.B. & TAKAKU, F. (eds). (1988). *The Status of Differentiation Therapy of Cancer*. Raven Press: New York.

WIEMANN, M., ALEXANDER, P. & CALABRESI, P. (1988). Combination differentiation therapy. In *The Status of Differentiation Therapy of Cancer*, S. Waxman, G.B., Rossi, F. Takaku (eds). pp. 299–314, Raven Press: New York.